# Gender Specificity and Local Socioeconomic Influence on Association of *GHR* fl/d3 Polymorphism With Growth and Metabolism in Children and Adolescents

**DOI:** 10.3389/fped.2022.546080

**Published:** 2022-03-23

**Authors:** Xiaotian Chen, Chunlan Liu, Song Yang, Yaming Yang, Yanchun Chen, Xianghai Zhao, Weiguang Zhu, Qihui Zhao, Chuan Ni, Xiangyuan Huang, Weili Yan, Chong Shen, Harvest F. Gu

**Affiliations:** ^1^Department of Clinical Epidemiology, Children's Hospital of Fudan University, Shanghai, China; ^2^Department of Epidemiology, School of Public Health, Nanjing Medical University, Nanjing, China; ^3^Department of Cardiology, People's Hospital of Yixing City, Affiliated Yixing People's Hospital of Jiangsu University, Yixing, China; ^4^Center for Disease Control and Prevention of Yixing City, Yixing, China; ^5^Center for Disease Control and Prevention of Suqian City, Suqian, China; ^6^School of Basic Medicine and Clinical Pharmacy, China Pharmaceutical University, Nanjing, China

**Keywords:** gender specificity, *GHR* fl/d3 polymorphism, growth hormone receptor, metabolism, socioeconomic levels

## Abstract

**Objective:**

Growth hormone receptor (*GHR*) mediates most GH biological actions. This study is aimed to evaluate whether *GHR* fl/d3 polymorphism contributes to the inter-individual variability of growth and metabolism in healthy children and adolescents.

**Methods:**

A total of 4,730 students aged 6-16 years from Yixing and Suqian City in China were included in this cross-sectional study. Height and body mass index (BMI) were transformed into the form of z-score corresponding to age and gender. Logistic regression was used to evaluate the associations of *GHR* fl/d3 polymorphism with height, BMI, metabolic traits, and hypertension by estimating the odds ratios (ORs) and 95% confidence intervals (CIs).

**Results:**

*GHR* d3 allele was inversely associated with overweight, total cholesterol (TC) and triglyceride (TG) levels (OR [95% CI] for overweight: 0.754 [0.593-0.959], *P* = 0.021; OR [95% CI] for TC: 0.744 [0.614-0.902], *P* = 0.003; OR [95% CI] for TG: 0.812 [0.654-0.998], *P* = 0.047). *GHR* d3 allele was associated with decreased odds of pre-hypertension in boys (OR [95% CI]: 0.791 [0.645-0.971], *P* = 0.025), but associated with increased odds of pre-hypertension and hypertension in girls (ORs [95% CIs]: 1.379 [1.106-1.719], *P* = 0.004; OR [95% CI]: 1.240 [1.013-1.519], *P* = 0.037). Interaction of *GHR* fl/d3 polymorphism with gender contributed to increased odds of pre-hypertension and hypertension (interactive ORs [95% CIs]: 1.735 [1.214-2.481], *P* = 0.003; OR [95% CI]: 1.509 [1.092-2.086], *P* = 0.013). Stratification analysis showed that the correlation tendencies of *GHR* fl/d3 polymorphism and BMI with age were different between two cities with discrepant economic development levels.

**Conclusion:**

*GHR* fl/d3 polymorphism is associated with growth, metabolism, and hypertension in children and adolescents with the gender specificity, and the genetic effect of *GHR* fl/d3 may be modified by the local socioeconomic levels.

## Introduction

Growth hormone receptor (GHR), a member of the cytokine receptor family, mediates the majority of growth hormone (GH) biological actions (1), which plays a major role in the postnatal linear growth and metabolic activity during childhood and adolescence (2). In humans, there are two isoforms of recognized *GHR* transcripts, full-length *GHR* (*GHR* fl) and exon 3-deleted *GHR* (*GHR* d3) (3). It is of importance to investigate the genetic effects of *GHR* fl/d3 polymorphism on inter-individual variability of growth, metabolic traits, and blood pressure (BP) in general children and adolescent population.

Children growth and development is one of the important issues in public health (4). GH exerts multiple complex biological effects on carbohydrate and lipid metabolism such as decrease in insulin sensitivity, ensuing glucose intolerance, and protein anabolism (5). All these actions are mediated through its interaction with GHR. A previous study conducted in 48 Turner syndrome girls showed that, compared with *GHR* fl/fl and fl/d3 genotype, *GHR* d3 allele homozygote was associated with a unique GH responsiveness and had a lower body mass index (BMI) level (6). Another case-control study with 262 morbidly obese subjects reported that *GHR* d3 allele carriers had higher BMI than *GHR* fl allele individuals, but GHR fl/d3 polymorphism was not associated with any other components of metabolic syndrome (7). Another cohort study included 385 community healthy subjects followed from birth to adult life observed that *GHR* d3 allele was not associated with body height or weight in young adults (8), while these findings were contrary to Gao et al.'s findings performed in 715 children (9). Moreover, Turgut et al. found that systolic blood pressure (SBP) was significantly increased in *GHR* d3/d3 genotype carriers compared to *GHR* fl/d3 subjects in acromegalic patients (*n* = 35) (10). Park et al. also performed a genetic study in acromegalic patients (*n* = 30), but no significant association of *GHR* fl/d3 polymorphism and SBP or diastolic blood pressure (DBP) was found (11).

Thereby, results from the previous studies are contradictive, and no socioeconomic issue has been considered in those analyses. Our study was aimed to evaluate whether *GHR* fl/d3 polymorphism was associated with growth, metabolic traits, and hypertension risk with a relatively large healthy children and adolescent population.

## Materials and Methods

### Study Population

This is a cross-section study that initially included a total of 6,160 subjects aged 6-16 years using a cluster sampling approach from Yixing and Suqian City in China (12, 13), which are, respectively, located in southern and northern Yangtze river (Supplementary Figure 1). Yixing City has a higher economy development level than Suqian City. One survey in Suqian City was conducted from October to November 2008 (*n* = 2,373), while another one in Yixing City was done in September 2014 (*n* = 3,787). All subjects were students from the local primary and junior high schools in a routine healthy examination program. The students aged under 6 years (*n* = 457), over 16 years (*n* = 40), and those with an incomplete physical examination record (*n* = 345) or refused to draw blood samples (*n* = 588) were excluded. Eventually, 4,730 children and adolescents were included in this study. The study protocols were approved by the Research Ethics Committee of Nanjing Medical University.

### Anthropometric Measurements

Anthropometric data including body height, weight, and BP were measured by standard methods (14). Body height was measured to the nearest 0.1 cm using a standard column stadiometer placed on a level ground. The students stood upright on bare feet, with heels together, and buttocks and back touching the meter rule. Body weight was measured to the nearest 0.1 kg using an electronic weightier, and the children were weighed wearing minimum clothing and without shoes. Both height and weight were measured twice. BMI value was calculated as weight (kg)/height squared (m^2^). Height and BMI were transformed to a z-score corresponding to age and gender (15), and normalized according to a nationwide study of height and weight standardized growth charts (16). All participants were classified into “height z-score < −1, −1 ≤ height z-score <1 and height z-score ≥1” groups, according to the World Health Organization (WHO) reference of height for different age and gender groups (17). In addition, subjects were divided into low body weight, overweight, and obesity groups based on the cut off points of the 3rd, 85th, and 95th centiles of BMI for age and gender (18, 19).

BP measurements were performed twice after resting for at least 5 min under a quiet position using the electronic sphygmomanometers (OMROM, HEM-7207, Dalian, China) according to a standard protocol (20). If the difference between these two measurements of either SBP or DBP was over 8 mmHg, an additional measurement was needed. Finally, the average values of two or three measurements of SBP and DBP were calculated for analysis. Likewise, BP value was transformed into BP z-score corresponding to age, gender, and height (21).

According to the reference values of “The Fourth Report on the Diagnosis, Evaluation, and Treatment of High Blood Pressure in Children and Adolescents” (22), the subjects were classified into normal BP, pre-hypertension, and hypertension groups. The normal BP group was defined as both SBP and DBP <90th percentile; average SBP and/or DBP at least 90th, but <95th, and/or SBP ≥ 120 or DBP ≥ 80 mmHg regardless of age, gender, and height was divided into the pre-hypertension group; the hypertension group was identified as the average SBP and/or DBP persistently above the 95th percentile, and/or SBP ≥ 140 or DBP ≥ 90 mmHg regardless of age, gender, and height.

### Biochemical Indices

Non-fasting peripheral venous blood was retained, and serum TC, TG, and GLU concentrations were measured by an automated biochemical profiling (Mindray BS-800, Shenzhen, China). Because there were no specifical cut-off values to identify the relative high levels of TC, TG, and GLU in children and adolescents, we calculated the value of the 90th percentile of TC, TG, and GLU to classify the students into normal group and relative high-level group in the association analyses.

### *GHR* fl/d3 Genotyping

DNA was extracted from the leukocyte of peripheral blood by using standard phenol-chloroform method (23). The polymerase chain reaction (PCR) method was used for genotyping *GHR* fl/d3 polymorphism in a 384-well plate. PCR was conducted in 10 μl of a reaction mixture containing about 10 ng of DNA sample, 1 μl reaction buffer, 1 μl MgCl_2_, 0.15 μl of platinum Taq DNA polymerase, and 100 *p*mol each of primer. This assay was carried out with primers G1, G2, and G3 (GenBank association no. AF155912) as follows (3): initial step of denaturation of 5 min at 95°C followed by 39 cycles consisting of 30 s at 95°C, 30 s at 64.8°C, 1 min 30 s at 72°C, followed by an extension period of 72°C for 10 min. Application of DNA fragments was analyzed by electrophoresis on a 1.5% agarose gel and visualized by ethidium bromide staining under ultraviolet (UV) light. The polymorphism detected by PCR was evident as a 592 bp fragment in the presence of the deletion (d3/d3) and a 935 bp product in the presence of the full-length fragment (fl/fl) of exon 3 in the *GHR* gene. Each sample was genotyped as *GHR* d3/d3, fl/d3, or fl/fl.

### Statistical Analysis

Continuous variables were expressed as mean ± standard deviation (SD) or as median (interquartile range). Independent sample *t*-test and analysis of variance (ANOVA) were used to analyze differences in continuous variables with normal distribution. Mann–Whitney *U*-test and Kruskal–Wallis test were used to analyze differences in continuous variables with non-Gaussian distribution. A chi-square test (χ^2^) was used for comparison of nominal variables between groups.

In the primary analyses, multivariate logistic regression model was used to estimate the associations of *GHR* fl/d3 polymorphism with height z-score levels (reference group: −1 ≤ height z-score < 1 group), obesity (reference group: normal weight group), and hypertension (reference group: normotensive group), while the binary logistic regression model was applied in the analysis of *GHR* fl/d3 polymorphism with metabolic traits using additive (fl/fl vs. fl/d3 vs. d3/d3), dominant (fl/fl + fl/d3 vs. d3/d3), and recessive (fl/fl vs. fl/d3 + d3/d3) models. Odds ratio (OR) and 95% confidence interval (CI) were calculated to quantify these associations. In the secondary analyses, we explored the modification effect of socioeconomic levels on the associations of *GHR* fl/d3 polymorphism with childhood height, BMI, metabolic traits, and hypertension through a stratification analysis. To evaluate the relationship of *GHR* fl/d3 polymorphism and BMI z-score among the subjects in different age groups, the consecutive tendencies of predicted BMI z-score by age stratified by gender and region were separately depicted by using the quadratic prediction plot. Besides, to explore the potential interaction between *GHR* fl/d3 polymorphism and gender on hypertension risk, Breslow-Day test for heterogeneity was performed and interaction figures were depicted using R-package “desctools” and “meta.” All the statistical analysis above was performed using Stata version 16.0 (Stata Corp LLC, College Station, TX) and R package (version 3.6.1). Two-tailed *P*-value <0.05 was defined to be statistically significant.

## Results

### Characteristics of the Participants

Demographic and clinical characteristics according to the genotypes of the *GHR* exon 3 polymorphisms were summarized in Table 1. A total of 4,730 students (2,465 boys and 2,265 girls) were included in the current study, with an average (SD) age of 10.83 (2.96) years. Totally, the numbers of *GHR* fl/fl, fl/d3, and d3/d3 genotype carriers were 3,287 (69.5%), 1,267 (26.8%), and 176 (3.7%), respectively. The percentage of *GHR* fl/fl, fl/d3, and d3/d3 carriers was significantly different among boys and girls with a *P*-value of 0.024. But no significant difference of age, height z-score, BMI z-score, SBP z-score, DBP z-score, TC, TG, and GLU was found among the three *GHR* genotypes.

**Table 1:** Demographic and clinical characteristics for the participants.

**Variable**	**Group**	**Total**	***GHR*** **fl/d3 polymorphism**			
			**fl/fl**	**fl/d3**	**d3/d3**	***P*-value**
Gender (*n*%)		4,730	3,287 (69.5)	1,267 (26.8)	176 (3.7)	
	Boys	2,465 (52.1)	1,691 (68.6)	665 (27.0)	109 (4.4)	0.024
	Girls	2,265 (47.9)	1,596 (70.5)	602 (26.6)	67 (2.9)	
Age (year)		10.83 ± 2.96	10.84 ± 2.95	10.83 ± 3.01	10.70 ± 3.03	0.836
Height z-score		0.11 (−0.72,0.86)	0.12 (−0.73,0.84)	0.12 (−0.7,0.89)	0.14 (−0.63,0.93)	0.858
BMI z-score		−0.66 (−0.19,0.45)	−0.18 (−0.66,0.46)	−0.19 (−0.68,0.45)	−0.21 (−0.69,0.45)	0.506
SBP z-score		0.52 (−0.37,1.31)	0.52 (−0.37,1.31)	0.49 (−0.42,1.29)	0.68 (−0.41,1.36)	0.386
DBP z-score		0.26 (−0.14,0.75)	0.27 (−0.14,0.76)	0.25 (−0.18,0.72)	0.31 (−0.03,0.82)	0.246
TC (mmol/L)		3.83 ± 0.57	3.76 ± 0.76	3.74 ± 0.74	3.66 ± 0.65	0.153
TG (mmol/L)		1.24 ± 0.74	1.07 ± 0.67	1.07 ± 0.63	1.01 ± 0.66	0.511
GLU (mmol/L)		4.51 ± 1.17	4.45 ± 1.10	4.43 ± 1.04	4.43 ± 1.08	0.920

*Continuous variables with normal distribution were expressed as means ± standard deviation*.

*Continuous variables with non-Gaussian distribution were expressed as median (interquartile range)*.

*BMI, body mass index; SBP, systolic blood pressure; DBP, diastolic blood pressure;*

*TC, total cholesterol; TG, triglyceride; GLU, glucose*.

### Associations of *GHR* fl/d3 Polymorphism With Height, BMI, and Hypertension

No statistical association of *GHR* d3 polymorphism and height z-score was observed in total subjects. In boys, the *GHR* d3 allele was significantly associated with lower height z-score, and ORs (95% CIs) for the additive model and dominant model were 1.221 (1.009-1.478) and 1.285 (1.016-1.624) with *P*-values of 0.040 and 0.036, respectively (Table 2). In girls, an inverse association was observed between *GHR* d3 allele and height z-score, and ORs (95% CIs) for the additive model and dominant model were 0.773 (0.614-0.972) and 0.749 (0.577-0.972) with *P*-values of 0.028 and 0.030, respectively.

**Table 2:** Association analysis of *GHR* fl/d3 polymorphism with height.

**Subject**	**Height z-score group**	**WT/HT/MT**	**Additive model**		**Dominant model**		**Recessive model**	
			**OR (95% CI)**	***P*-value**	**OR (95% CI)**	***P*-value**	**OR (95% CI)**	***P*-value**
**Total subjects**
	−1~1	2,117/810/109	Reference		Reference		Reference	
	< −1	529/202/28	1.004 (0.868-1.162)	0.957	1.002 (0.842-1.191)	0.985	1.024 (0.671–1.563)	0.912
	≥1	641/255/39	1.059 (0.928-1.209)	0.396	1.057 (0.902-1.238)	0.495	1.164 (0.801–1.690)	0.426
**Boys**								
	−1~1	1,049/385/60	Reference		Reference		Reference	
	< −1	255/119/20	1.221 (1.009-1.478)	0.040	1.285 (1.016-1.624)	0.036	1.272 (0.757–2.137)	0.364
	≥1	387/161/29	1.138 (0.962-1.348)	0.132	1.157 (0.941-1.422)	0.166	1.259 (0.799–1.982)	0.321
**Girls**								
	−1~1	1,068/425/49	Reference		Reference		Reference	
	< −1	274/83/8	0.773 (0.614-0.972)	0.028	0.749 (0.577-0.972)	0.030	0.680 (0.319–1.449)	0.318
	≥1	254/94/10	0.929 (0.746-1.156)	0.508	0.923 (0.717-1.188)	0.534	0.872 (0.437-1.739)	0.697

*WT, fl /fl genotype; HT, fl /d3 genotype; MT, d3/d3 genotype; OR, odds ratio; CI, confidence interval*.

*GHR* d3 allele variation was associated with decreased odds of overweight in the entire population and ORs (95% CIs) for the additive model and dominant model were 0.754 (0.593-0.959) and 0.738 (0.561-0.971) with *P*-values of 0.021 and 0.030, respectively (Table 3). In boys, similar association of *GHR* d3 allele with overweight was observed (OR [95% CI] for the additive model was 0.717 [0.515-0.998], *P* = 0.049), while no significant association was found in girls.

**Table 3:** Association analysis of *GHR* fl/d3 polymorphism with BMI.

**Subject**	**BMI group**	**WT/HT/MT**	**Additive model**		**Dominant model**		**Recessive model**	
			**OR (95% CI)**	***P-*value**	**OR (95% CI)**	***P*-value**	**OR (95% CI)**	***P-*value**
**Total subjects**
	Normal weight	2,001/786/103	Reference		Reference		Reference	
	Low weight	826/323/56	1.057 (0.937-1.193)	0.369	1.030 (0.891-1.191)	0.692	0.135 (0.943-1.836)	0.107
	Overweight	225/68/6	0.754 (0.593-0.959)	0.021	0.738 (0.561-0.971)	0.030	0.553 (0.240-1.270)	0.163
	Obesity	235/90/11	0.965 (0.783-1.189)	0.736	0.965 (0.754-1.234)	0.774	0.914 (0.485-1.720)	0.779
**Boys**								
	Normal weight	1,071/418/65	Reference		Reference		Reference	
	Low weight	381/161/35	1.153 (0.978-1.360)	0.090	1.136 (0.927-1.392)	0.218	1.486 (0.973-2.270)	0.067
	Overweight	118/37/2	0.717 (0.515-0.998)	0.049	0.730 (0.500-1.065)	0.103	0.297 (0.072-1.225)	0.093
	Obesity	121/49/7	1.008 (0.764-1.33)	0.953	1.022 (0.731-1.429)	0.897	0.948 (0.427-2.101)	0.895
**Girls**								
	Normal weight	930/368/38	Reference		Reference		Reference	
	Low weight	455/162/21	0.971 (0.812-1.162)	0.749	0.940 (0.764-1.158)	0.563	1.165 (0.678-2.003)	0.580
	Overweight	107/31/4	0.801 (0.565-1.136)	0.213	0.748 (0.502-1.115)	0.154	0.976 (0.343-2.777)	0.964
	Obesity	114/41/4	0.914 (0.665-1.255)	0.914	0.903 (0.627-1.300)	0.582	0.869 (0.306-2.468)	0.792

*WT, fl /fl genotype; HT, fl /d3 genotype; MT, d3/d3 genotype; OR, odds ratio; CI, confidence interval*.

No statistical associations of *GHR* d3 polymorphism with pre-hypertension or hypertension were observed in total subjects. Boys with *GHR* d3 allele were associated with decreased odds of pre-hypertension, and ORs (95% CIs) for the additive model and dominant model were 0.791 (0.645-0.971) and 0.777 (0.609-0.990) with *P*-values of 0.025 and 0.042, respectively (Table 4). In girls, *GHR* d3 allele was associated with increased odds of pre-hypertension and hypertension, and ORs (95% CIs) for the additive model were 1.379 (1.106-1.719) and 1.240 (1.013-1.519) with *P*-values of 0.004 and 0.037, respectively.

**Table 4:** Association analysis of *GHR* fl/d3 polymorphism with pre-hypertension and hypertension.

**Subject**	**Blood pressure group**	**WT/HT/MT**	**Additive model**		**Dominant model**		**Recessive model**	
			**OR (95% CI)**	***P*-value**	**OR (95% CI)**	***P*-value**	**OR (95% CI)**	***P*-value**
**Total subjects**								
	Normotensive	2,166/846/109	Reference		Reference		Reference	
	Pre-hypertension	487/187/28	1.018 (0.877-1.181)	0.819	1.003 (0.841-1.199)	0.970	1.133 (0.742-1.731)	0.562
	Hypertension	634/234/39	1.010 (0.883-1.156)	0.881	0.979 (0.833-1.150)	0.793	1.226 (0.844-1.781)	0.285
**Boys**								
	Normotensive	1,045/444/75	Reference		Reference		Reference	
	Pre-hypertension	287/99/12	0.791 (0.645-0.971)	0.025	0.777 (0.609-0.990)	0.042	0.610 (0.328-1.133)	0.118
	Hypertension	359/122/22	0.851 (0.709-1.021)	0.082	0.805 (0.646-1.004)	0.054	0.897 (0.552-1.459)	0.662
**Girls**								
	Normotensive	1,121/402/34	Reference		Reference		Reference	
	Pre-hypertension	200/88/16	1.379 (1.106-1.719)	0.004	1.347 (1.037-1.751)	0.026	2.456 (1.338-4.508)	0.004
	Hypertension	275/112/17	1.240 (1.013-1.519)	0.037	1.215 (0.959-1.541)	0.107	1.942 (1.073-3.513)	0.028

*WT, fl /fl genotype; HT, fl /d3 genotype; MT, d3/d3 genotype; OR, odds ratio; CI, confidence interval*.

### Associations of *GHR* fl/d3 Polymorphism With Metabolic Traits

In total population, *GHR* d3 allele was inversely associated with higher TC level, and ORs (95% CIs) of additive, dominant, and recessive models were 0.744 (0.614-0.902), 0.757 (0.608-0.942), and 0.372 (0.173-0.799) with *P*-values of 0.003, 0.013, and 0.011, respectively (Table 5). The significant association between *GHR* d3 allele and higher TC level was further observed in the subgroup of boys and girls, and ORs (95% CIs) of additive model were 0.742 (0.565-0.973) and 0.747 (0.568-0.982) with *P*-values of 0.031 and 0.037, respectively. *GHR* d3 allele was inversely associated with higher TG level and OR (95% CI) was 0.812 (0.654-0.998) with a *P*-value of 0.047.

**Table 5:** Association analysis of *GHR* fl/d3 polymorphism with metabolic traits.

**Subject**	**Group**	**WT/HT/MT**	**Additive model**		**Dominant model**		**Recessive model**	
			**OR (95% CI)**	***P*-value**	**OR (95% CI)**	***P*-value**	**OR (95% CI)**	***P*-value**
**Total subjects**								
	Normal group	2,957/1130/161	Reference		Reference		Reference	
	High GLU	330/137/15	1.016 (0.855–1.209)	0.854*	1.054 (0.859-1.295)	0.612*	0.824 (0.479-1.416)	0.483*
	Normal group	2,936/1154/169	Reference		Reference		Reference	
	High TC	351/113/7	0.744 (0.614-0.902)	0.003*	0.757 (0.608-0.942)	0.013*	0.372 (0.173-0.799)	0.011*
	Normal group	2,939/1156/161	Reference		Reference		Reference	
	High TG	348/111/15	0.845 (0.702-1.016)	0.074*	0.812 (0.654-0.998)	0.047*	0.853 (0.496-1.469)	0.567*
**Boys**								
	Normal group	1,512/596/100	Reference		Reference		Reference	
	High GLU	179/69/9	0.935 (0.738-1.183)	0.574^#^	0.948 (0.715-1.257)	0.711^#^	0.777 (0.386-1.562)	0.479^#^
	Normal group	1,528/612/104	Reference		Reference		Reference	
	High TC	163/53/5	0.742 (0.565-0.973)	0.031^#^	0.735 (0.536-1.008)	0.056^#^	0.479 (0.193-1.189)	0.112^#^
	Normal group	1,521/607/102	Reference		Reference		Reference	
	High TG	170/58/7	0.830 (0.659-1.125)	0.154^#^	0.824 (0.609-1.115)	0.210^#^	0.650 (0.296-1.426)	0.282^#^
**Girls**								
	Normal group	1,445/534/61	Reference		Reference		Reference	
	High GLU	151/68/6	1.128 (0.873-1.459)	0.357^#^	1.191 (0.883-1.607)	0.251^#^	0.906 (0.384-2.134)	0.906^#^
	Normal group	1,408/542/65	Reference		Reference		Reference	
	High TC	188/60/2	0.747 (0.568-0.982)	0.037^#^	0.777 (0.574-1.054)	0.105^#^	0.239 (0.058-0.983)	0.047^#^
	Normal group	1,418/549/59	Reference		Reference		Reference	
	High TG	178/53/8	0.861 (0.659-1.125)	0.273^#^	0.801 (0.588-1.090)	0.157^#^	1.165 (0.547-2.482)	0.692^#^

* *Adjusted for age and gender*;

# *adjusted for age; WT, fl /fl genotype; HT, fl /d3 genotype; MT, d3/d3 genotype; OR, odds ratio; CI, confidence interval*.

### Stratification Analysis

Stratification analysis of *GHR* fl/d3 polymorphism with height, BMI, hypertension, and metabolic traits by region was performed (Supplementary Tables 1–4). *GHR* d3 variation was associated with increased odds of low BMI levels in Suqian boys and OR (95% CI) of additive model was 1.323 (1.034-1.693) with *P*-value of 0.026. *GHR* d3 allele was associated with reduced odds of overweight in Suqian girls, and OR (95% CI) for the dominant model was 0.456 (0.209-0.996) with *P* of 0.049. No significant associations between *GHR* d3 variation and height levels were observed among Yixing and Suqian students.

Compared with *GHR* fl/fl and fl/d3 genotypes, a unimodal shape of *GHR* d3/d3 on BMI z-score by age was visually observed in boys and girls (Supplementary Figure 2). This tendency of *GHR* d3/d3 on BMI was further displayed in Yixing and Suqian city (Supplementary Figure 3). After we stratified by region and gender, a similar effect of *GHR* fl/d3 and d3/d3 genotypes on BMI was observed in Yixing boys, while the effect of *GHR* d3/d3 on BMI was inversed in Yixing girls. For *GHR* fl/fl and fl/d3 carriers in Suqian, BMI z-score was decreased with the increase of age in boys, which was contrary to the d3/d3 genotype. In Suqian girls, the fluctuating range of *GHR* d3/d3 on BMI was obviously enlarged and its peak approximately appeared in the 10- or 11-year groups (Supplementary Figure 4).

*GHR* d3 allele was associated with reduced odds of pre-hypertension and hypertension in Yixing boys (ORs [95% CIs] for the additive model and dominant model: 0.750 [0.585-0.962], *P* = 0.023; 0.757 [0.587-0.976], *P* = 0.031; Supplementary Table 3). Conversely, *GHR* d3 allele was associated with increased odds of pre-hypertension and hypertension in Yixing girls (ORs [95% CIs] for the additive model: 1.366 [1.055-1.770], *P* = 0.018; 1.273 [1.009-1.606], *P* = 0.042). No significant associations were found between *GHR* fl/d3 polymorphism and hypertension in Suqian City.

### Interaction Between *GHR* fl/d3 Polymorphism and Gender on Hypertension

Breslow-Day test showed significant interactive effects of *GHR* fl/d3 polymorphism and gender on pre-hypertension and hypertension risk existed, and the *P*-values of the homogeneity test were 0.002 and 0.013, respectively (Supplementary Figure 5). *GHR* fl/d3 dominant model had positive multiplicative interactions on pre-hypertension and hypertension (interactive ORs [95% CIs]: 1.735 [1.214-2.481], *P* = 0.003; 1.509 [1.092-2.086], *P* = 0.013; Supplementary Tables 5, 6).

## Discussion

In the current study, we investigated the associations of *GHR* fl/d3 polymorphism with height, BMI, hypertension, and metabolic traits in healthy children and adolescents with the largest sample size. We observed the multiplicative interaction of *GHR* fl/d3 polymorphism with gender contributing to increased odds of pre-hypertension and hypertension and found that *GHR* d3 allele was likely to have a protective effect against high TC level. Furthermore, genetic effect of this polymorphism on BMI was inconsistent in different age groups, which may be modified by the local socioeconomic levels.

Hypertension in children and adolescents has been recognized as a challenge (24), to which genetic polymorphism and their interaction with environmental factors may contribute (25). Lack of GHR signaling causes a reduction in SBP and plasma renin levels as well as an increase in aortic eNOS expression (26). Animal studies showed *GHR* knockout mice led to a 25% reduction in SBP compared with wild-type mice (26). In the current study, we assessed the associations of this polymorphism with hypertension status in general childhood population. *GHR* d3 allele is found to be associated with decreased odds of pre-hypertension and hypertension in boys, while increased odds of pre-hypertension and hypertension in girls was observed. Furthermore, interaction between *GHR* fl/d3 polymorphism and gender on pre-hypertension and hypertension was first illustrated, suggesting genetic effects of this polymorphism may have a gender specificity.

Socioeconomy is a conspicuous environmental factor that affects physical growth in children. Yixing and Suqian are two cities located in south and north of the Yangtze River, China. Traditionally and currently, the socioeconomic level in Yixing is significantly higher than that in Suqian (Supplementary Figure 1). These two cities are different not only in geography but also in social developing stages from history until now. We thus separately analyzed the associations of *GHR* fl/d3 polymorphism with growth and metabolic traits in children and adolescents from Yixing and Suqian Cities. Data demonstrated that the carriers with *GHR* d3 allele are associated with low BMI levels in Suqian children. A similar trend of this association in Yixing children was observed but not statistically significant. These findings are consistent with the previous studies (9, 27). The association of *GHR* d3 allele with low BMI is speculated that biological function of this allele may increase lipolytic effect (28), which is helpful in keeping a relatively low body fat mass composition (29). We have further depicted the different *GHR* genotypes of BMI fluctuation among 6-16 years age groups in boys and girls. The large swing of BMI variation among *GHR* fl/d3 genotypes with age was displayed in Yixing and Suqian, indicating that genetic effects of *GHR* fl/d3 polymorphism on childhood BMI could be modified by the local socioeconomic levels.

A previous study has reported that *GHR* d3 allele had a 30% higher bioactivity than *GHR* fl allele (27). Data from the current study demonstrated that *GHR* d3/d3 on BMI had a larger fluctuation with age than fl/fl and fl/d3 genotypes. Together with the implication from the previous study (27), our data consistently suggest that *GHR* d3 allele may have a vital role on BMI in children and adolescents. A unimodal peak of *GHR* d3/d3 on BMI with age approximately appeared at 10 or 11 years group, indicating the relationship between *GHR* fl/d3 polymorphism and BMI is significantly different at puberty in boys and girls. Stratified by region and gender, compared with Suqian City, the effect of *GHR* d3/d3 genotype on BMI was relatively moderated in Yixing City. This finding may also support our postulate that socioeconomy modifies the genetic effect of *GHR* fl/d3 polymorphism on BMI. Furthermore, the direction of association of *GHR* d3 allele on height levels was opposite in boys and girls, further investigations to understand how puberty interacts with GH in the regulation of BMI and height could be taken into consideration.

Previous studies showed that no significant difference of lipids profile in *GHR* fl/d3 genotype carriers in children and adolescents was observed (7, 8). The relatively small sample sizes of those studies may be a likely reason for the null association. Whether *GHR* fl/d3 polymorphism associates with metabolic traits is still not well investigated. Our study presented *GHR* d3 allele may have a negative relationship with higher levels of TC and TG, suggesting that the *GHR* d3 allele may have a protective function against the abnormal lipid metabolism. One possible explanation is that the bioactivity of GHR regulated the carbohydrate and lipid metabolism via several downstream insulin signaling events (30).

Several limitations existed in this study. First, potential confounding factors, such as calorie intake and physical exercise, that may introduce bias by modifying the associations between *GHR* fl/d3 polymorphism, BMI, metabolic traits, and hypertension were not examined. Second, this population with Han nationality were from east China, the generalizability of our findings to other regions needs further validation. Third, in this study, the distribution of *GHR* fl/d3 polymorphism was deviated from Hardy-Weinberg equilibrium (HWE) in the total population. Nevertheless, we excluded the genotyping error by checking the raw data of the genotyping results among each batch, where no large deviation of *GHR* fl/d3 frequency among each batch was found. Thus, the interpretation of our findings could be convincing to some extent in the case of departure from HWE. Moreover, the exploratory nature of our study without a prespecified power calculation precludes us from drawing any confirmative conclusions.

In conclusion, the current study demonstrated that *GHR* fl/d3 polymorphism was associated with BMI, metabolism, and hypertension in children and adolescents, which may be modified by local socioeconomic levels.

## Data Availability Statement

The datasets generated for this study are available on request to the corresponding author.

## Ethics Statement

The studies involving human participants were reviewed and approved by Research Ethics Committee of Nanjing Medical University. Written informed consent to participate in this study was provided by the participants' legal guardian/next of kin.

## Author Contributions

HG and CS proposed and designed the study. CL, XC, SY, CN, QZ, YY, YC, XZ, WZ, and QZ collected the data. CL, XC, XH, SY, and CS carried out all analyses. CL and XC wrote the draft. WY, CS, and HG edited the manuscript. All authors contributed to data interpretation, discussion, and revision of the article.

## Funding

This work was supported by the National Natural Science Foundation of China (Grant Nos. 81872686 and 81573232), Jiangsu Provincial Fourth 333 Project, and the Priority Academic Program for the Development of Jiangsu Higher Education Institutions (Public Health and Preventive Medicine). The funders had no role in the design and conduct of the study, collection, management, analysis, interpretation of the data, preparation, review, or approval of the manuscript, and decision to submit the manuscript for publication.

## Conflict of Interest

The authors declare that the research was conducted in the absence of any commercial or financial relationships that could be construed as a potential conflict of interest.

## Publisher's Note

All claims expressed in this article are solely those of the authors and do not necessarily represent those of their affiliated organizations, or those of the publisher, the editors and the reviewers. Any product that may be evaluated in this article, or claim that may be made by its manufacturer, is not guaranteed or endorsed by the publisher.

## References

[1] Laron Z. Natural history of the classical form of primary growth hormone (GH) resistance (Laron syndrome) *J Pediatr Endocrinol Metab*. (1999) 12(Suppl 1):231-49. 10698588

[2] Veldhuis JD Roemmich JN Richmond EJ Rogol AD Lovejoy JC Sheffield-Moore M. Endocrine control of body composition in infancy, childhood, and puberty. Endocr Rev. (2005) 26:114-46. 10.1210/er.2003-0038 15689575

[3] Pantel J Machinis K Sobrier ML Duquesnoy P Goossens M Amselem S. Species-specific alternative splice mimicry at the growth hormone receptor locus revealed by the lineage of retroelements during primate evolution. J Biol Chem. (2000) 275:18664-9. 10.1074/jbc.M001615200 10764769

[4] Winbery S Blaho K. Pediatric growth and development. Optom Clin. (1996) 5:35–59. 8837126

[5] Moller N Jorgensen JO. Effects of growth hormone on glucose, lipid, and protein metabolism in human subjects. Endocr Rev. (2009) 30:152–77. 10.1210/er.2008-0027 19240267

[6] Binder G Trebar B Baur F Schweizer R Ranke MB. Homozygosity of the d3-growth hormone receptor polymorphism is associated with a high total effect of GH on growth and a low BMI in girls with Turner syndrome. Clin Endocrinol. (2008) 68:567–72. 10.1111/j.1365-2265.2007.03090.x 17973940

[7] Espinosa E Salame L Marrero-Rodriguez D Romero-Nieves AM Cuenca D Castelan-Martinez OD. Expression of the growth hormone receptor isoforms and its correlation with the metabolic profile in morbidly obese subjects. Endocrine. (2019) 63:573–81. 10.1007/s12020-018-1794-y 30361972

[8] Martins CS Fernandesrosa FL Espineira AR de Souza RM De CM Barbieri MA. The growth hormone receptor exon 3 polymorphism is not associated with height or metabolic traits in healthy young adults. Growth Hormone IGF Res. (2014) 24:123–9. 10.1016/j.ghir.2014.04.005 24893921

[9] Gao LL Zheng ZQ Cao LF Shen SX Yang Y Zhao ZH. The growth hormone receptor (GHR) exon 3 polymorphism and its correlation with metabolic profiles in obese Chinese children. Pediatr Diabetes. (2011) 12:429–34. 10.1111/j.1399-5448.2010.00747.x 21470351

[10] Turgut S Akin F Ayada C Topsakal S Yerlikaya E Turgut G. The growth hormone receptor polymorphism in patients with acromegaly: relationship to BMI and glucose metabolism. Pituitary. (2012) 15:374–9. 10.1007/s11102-011-0329-9 21744231

[11] Park H Hwang I Seo J Kim S Seo H Lee I. Association between the growth hormone receptor exon 3 polymorphism and metabolic factors in Korean patients with acromegaly. Endocrinol Metab. (2015) 30:312–7. 10.3803/EnM.2015.30.3.312 25559716 PMC4595356

[12] Guo D Shen C Chen Y Yang S Wang L Jin Y. Polymorphisms of the TGFBRAP1 gene in relation to blood pressure variability and plasma TGF-beta1. Clin Exp Hypertens. (2015) 37:420–5. 10.3109/10641963.2015.1013113 25856002

[13] Chen XT Yang S Yang YM Zhao HL Chen YC Zhao XH. Exploring the relationship of peripheral total bilirubin, red blood cell, and hemoglobin with blood pressure during childhood and adolescence. J Pediatr. (2018) 94:532–38. 10.1016/j.jpedp.2017.11.006 29107800

[14] Lohman TJ Roache AF Martorell R. Anthropometric standardization reference manual. Med Sci Sports Exerc. (1991) 24:952. 10.1249/00005768-199208000-00020

[15] Kuczmarski RJ Ogden CL Guo SS Grummerstrawn LM Flegal KM Mei Z. 2000 CDC growth charts for the United States: methods and development. Vital Health Statistics 11. (2002) 246:1. 12043359

[16] Li H Ji CY Zong XN Zhang YQ. [Height and weight standardized growth charts for Chinese children and adolescents aged 0 to 18 years]. Zhonghua Er Ke Za Zhi. (2009) 47:487–92. 10.3760/cma.j.issn.0578-1310.2009.07.003 19951507

[17] Listed N. Physical Status: The Use and Interpretation of Anthropometry. Report of a WHO Expert Committee. Geneva: World Health Organization (1995). 8594834

[18] Barlow SE Dietz WH. Obesity evaluation and treatment: expert Committee recommendations. The Maternal and Child Health Bureau, Health Resources and Services Administration and the Department of Health and Human Services. Pediatrics. (1998) 102:E29. 9724677 10.1542/peds.102.3.e29

[19] Cole TJ Flegal KM Nicholls D Jackson AA. Body mass index cut offs to define thinness in children and adolescents: international survey. BMJ. (2007) 335:194. 10.1136/bmj.39238.399444.55 17591624 PMC1934447

[20] Perloff D Grim C Flack J Frohlich ED Hill M Mcdonald M. Human blood pressure determination by sphygmomanometry. Circulation. (1993) 88:2460–70. 10.1161/01.CIR.88.5.2460 8222141

[21] Yan W Liu F Li X Wu L Zhang Y Cheng Y. Blood pressure percentiles by age and height for non-overweight Chinese children and adolescents: analysis of the china health and nutrition surveys 1991–2009. BMC Pediatrics. (2013) 13:195. 10.1186/1471-2431-13-195 24274040 PMC4222552

[22] National High Blood Pressure Education Program Working Group on High Blood Pressure in Children and Adolescents. The fourth report on the diagnosis, evaluation, and treatment of high blood pressure in children and adolescents. Pediatrics. (2004) 114:555. 10.1542/peds.114.2.S2.555 15286277

[23] Sambrook J Russell DW. Isolation of high-molecular-weight DNA from mammalian cells using formamide, chapter 6.13. Cold Spring Harb Protoc. (2006). 10.1101/pdb.prot3225 22485258

[24] Danaei G Finucane MM Lin JK Singh GM Paciorek CJ Cowan MJ. National, regional, and global trends in systolic blood pressure since 1980: systematic analysis of health examination surveys and epidemiological studies with 786 country-years and 5.4 million participants. Lancet. (2011) 377:568–77. 10.1016/S0140-6736(10)62036-3 21295844

[25] Waken RJ de Las Fuentes L Rao DC. A review of the genetics of hypertension with a focus on gene-environment interactions. Curr Hypertens Rep. (2017) 19:23. 10.1007/s11906-017-0718-1 28283927 PMC5647656

[26] Egecioglu E Andersson IJ Bollano E Palsdottir V Gabrielsson BG Kopchick JJ. Growth hormone receptor deficiency in mice results in reduced systolic blood pressure and plasma renin, increased aortic eNOS expression, and altered cardiovascular structure and function. Am J Physiol Endocrinol Metab. (2007) 292:E1418-25. 10.1152/ajpendo.00335.2006 17244725

[27] Dos Santos C Essioux L Teinturier C Tauber M Goffin V Bougneres P. A common polymorphism of the growth hormone receptor is associated with increased responsiveness to growth hormone. Nat Genet. (2004) 36:720–4. 10.1038/ng1379 15208626

[28] Berryman DE List EO. Growth hormone's effect on adipose tissue: quality versus quantity. Int J Mol Sci. (2017) 18:1621. 10.3390/ijms18081621 28933734 PMC5578013

[29] van der Klaauw AA van der Straaten T Baak-Pablo R Biermasz NR Guchelaar HJ Pereira AM. Influence of the d3-growth hormone (GH) receptor isoform on short-term and long-term treatment response to GH replacement in GH-deficient adults. J Clin Endocrinol Metab. (2008) 93:2828–34. 10.1210/jc.2007-2728 18397980

[30] Dominici FP Turyn D. Growth hormone-induced alterations in the insulin-signaling system. Exp Biol Med. (2002) 227:149–57. 10.1177/153537020222700301 11856812

