# Supplementary material for: Gender Specificity and Local Socioeconomic Influence on Association of GHR fl/d3 Polymorphism With Growth and Metabolism in Children and Adolescents
Source: Front Pediatr. 2022 Mar 23;10:546080. doi: 10.3389/fped.2022.546080 (PMC8984194; doi:10.3389/fped.2022.546080)
Supplement: Supplementary file 1 [file Data_Sheet_1.doc]

**Supplementary files**

**Gender Specificity and Local Socioeconomic Influence on Association of *GHR* fl/d3 Polymorphism with Growth** **and Metabolism in Children and Adolescents**

***Short title****: GHR fl/d3 in children and adolescents*


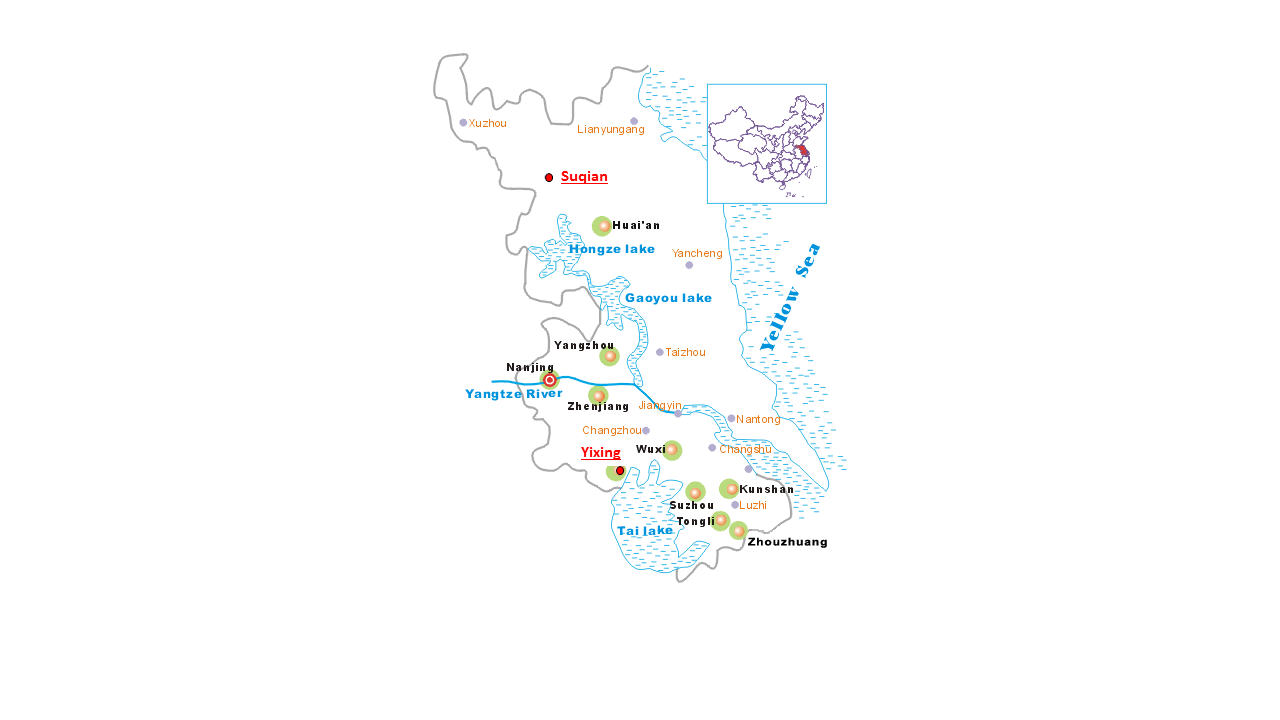


**Figure S1.** Geographical location of Yixing and Suqian cities in China. Yangtze is the longest river in China and divided this country by southern and northern parts. Both Yixing and Suqian cities are located in Jiangsu province but respectively in southern and northern of Yangtze river. According to the developing stages of society and economy, GDP in Yixing is much higher than what in Suqian.


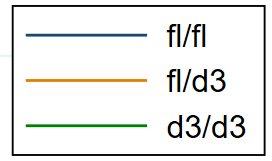

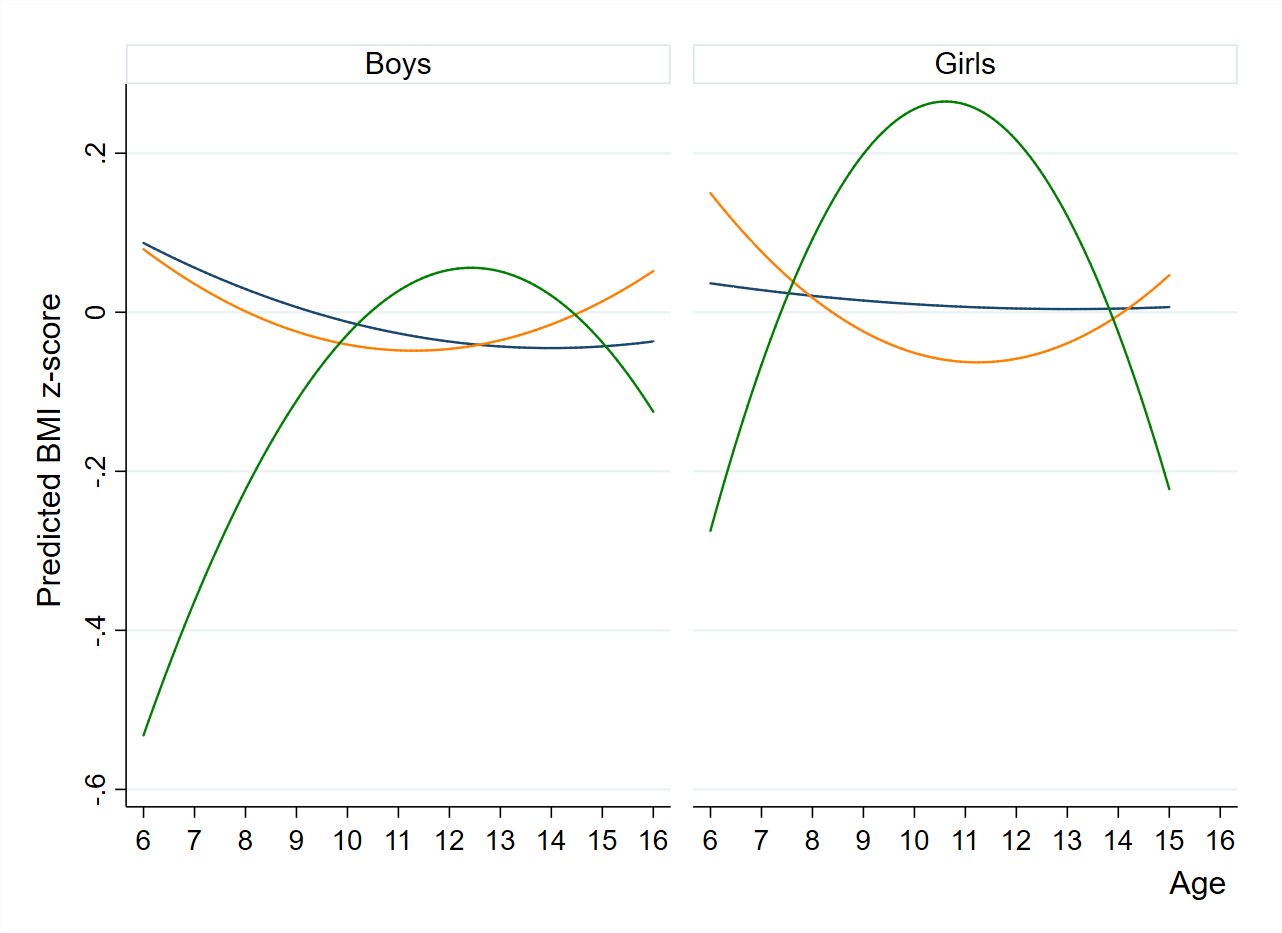


**Figure S2.** Consecutive tendencies of predicted BMI z-score by the age according to the genotypes of *GHR* fl/d3 polymorphism in boys and girls.


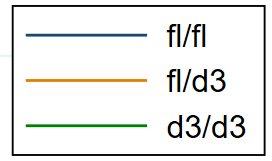

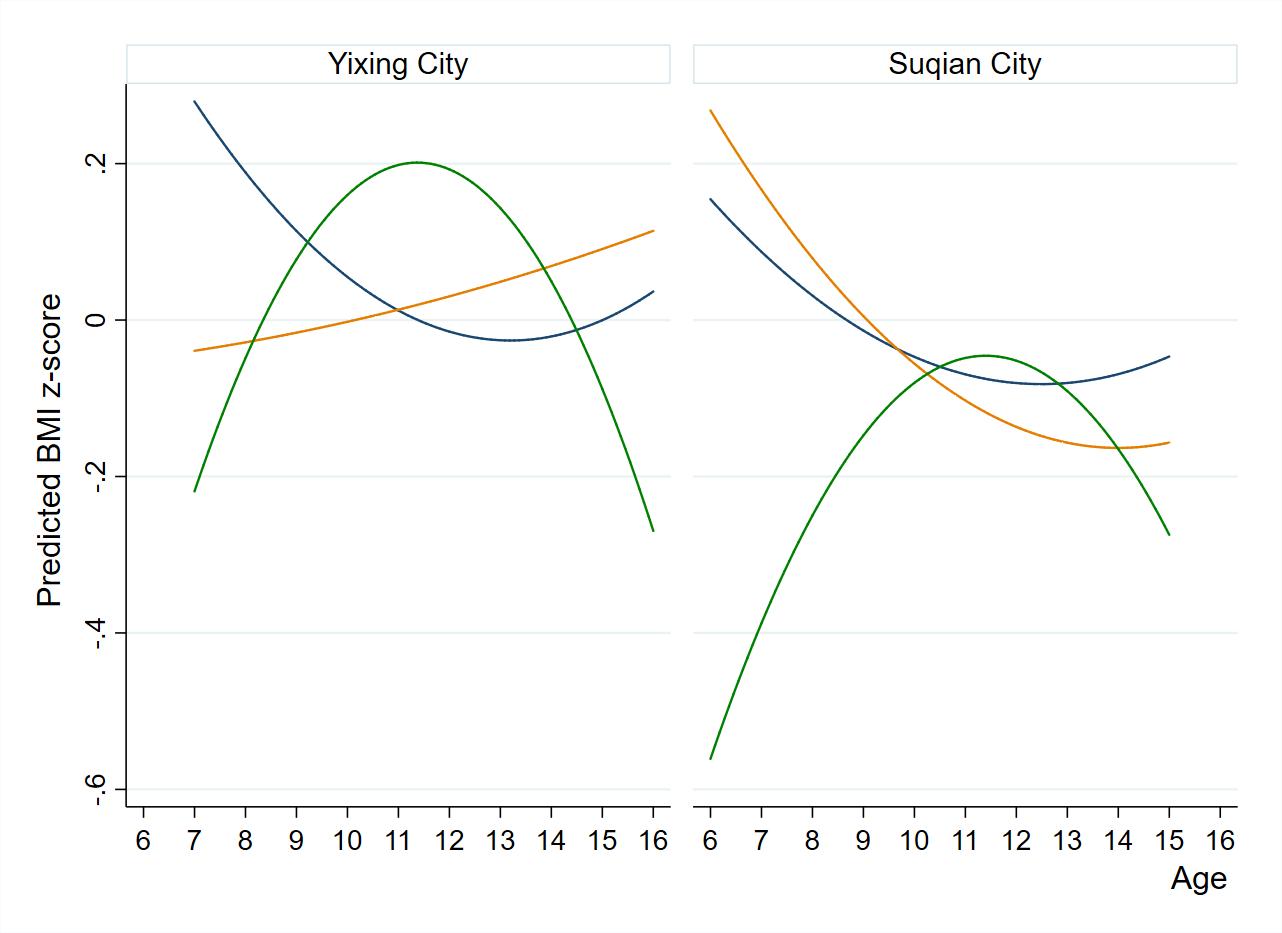


**Figure S3.** Consecutive tendencies of predicted BMI z-score by age according to the genotypes of *GHR* fl/d3 polymorphism among children and adolescents in Yixing and Suqian.


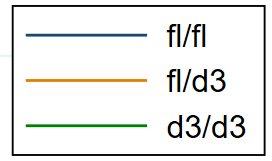




**Figure S4.** Consecutive tendency of predicted BMI-z score by age and gender according to the genotypes of *GHR* fl/d3 polymorphism among boys and girls in Yixing (A) and Suqian (B).


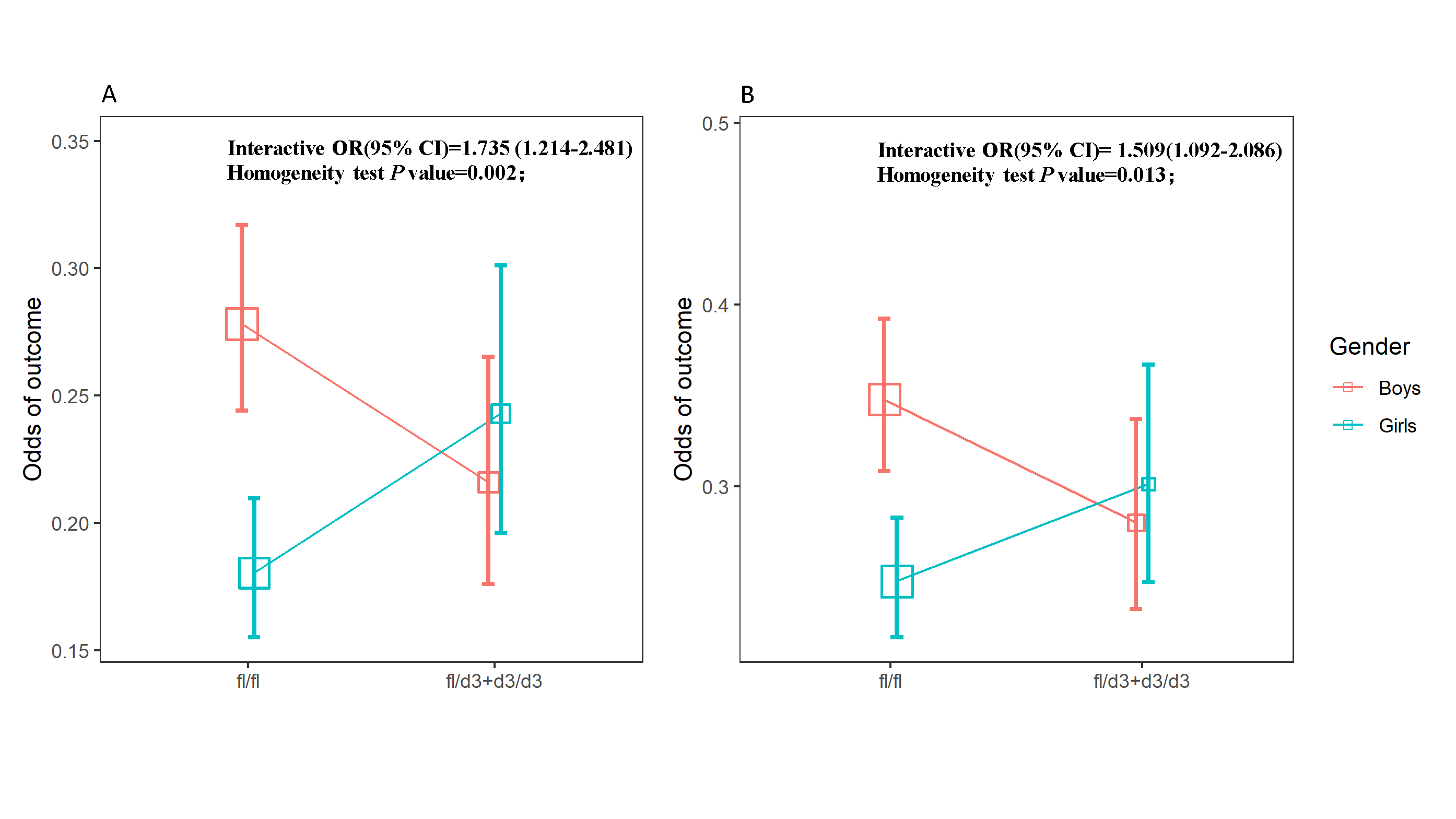


**Figure S5.** Interaction of the *GHR* fl/d3 polymorphism with gender on pre-hypertension (A) and hypertension (B) among children and adolescents.

| **Table S1. Stratification analysis of *GHR* fl/d3 polymorphism with height by region** | | | | | | | | | | | |
| --- | --- | --- | --- | --- | --- | --- | --- | --- | --- | --- | --- |
| Region | Group | Height-z score | WT/HT/MT | Additive model | |  | Dominant model | |  | Recessive model | |
|  |  |  |  | OR (95% CI) | *P*-value |  | OR (95% CI) | *P*-value |  | OR (95% CI) | *P*-value |
| Yixing | Boys | ﹣1~1 | 562/216/27 | Reference |  |  | Reference |  |  | Reference |  |
|  |  | <﹣1 | 236/108/18 | 1.222(0.981-1.521) | 0.074 |  | 1.238(0.951-1.612) | 0.112 |  | 1.498(0.814-2.756) | 0.194 |
|  |  | ＞1 | 232/100/15 | 1.138(0.908-1.427) | 0.261 |  | 1.150(0.878-1.506) | 0.311 |  | 1.294(0.679-2.463) | 0.434 |
|  | Girls | ﹣1~1 | 655/261/33 | Reference |  |  | Reference |  |  | Reference |  |
|  |  | ＜﹣1 | 254/79/8 | 0.784(0.614-0.999) | 0.050 |  | 0.762(0.576-1.008) | 0.057 |  | 0.664(0.304-1.452) | 0.305 |
|  |  | ＞1 | 98/39/6 | 1.040(0.756-1.430) | 0.812 |  | 1.022(0.700-1.493) | 0.911 |  | 1.210(0.498-2.942) | 0.674 |
| Suqian | Boys | ﹣1~1 | 487/169/33 | Reference |  |  | Reference |  |  | Reference |  |
|  |  | ＜﹣1 | 19/11/2 | 1.412(0.811-2.460) | 0.222 |  | 1.643(0.796-3.390) | 0.179 |  | 1.321(0.303-5.766) | 0.711 |
|  |  | ＞1 | 155/61/14 | 1.142(0.886-1.472) | 0.305 |  | 1.162(0.843-1.601) | 0.360 |  | 1.285(0.675-2.445) | 0.446 |
|  | Girls | ﹣1~1 | 413/164/16 | Reference |  |  | Reference |  |  | Reference |  |
|  |  | ＜﹣1 | 20/4/0 | 0.458(0.162-1.293) | 0.140 |  | 0.460(0.155-1.366) | 0.162 |  | - | - |
|  |  | ＞1 | 156/55/4 | 0.868(0.637-1.183) | 0.370 |  | 0.871(0.615-1.232) | 0.434 |  | 0.681(0.225-2.061) | 0.497 |

WT, *GHR* fl/fl genotype; HT, *GHR* fl/d3 genotype; MT, *GHR* d3/d3 genotype; OR, odds ratio; CI, confidence interval.

**Table S2. Stratification analysis of *GHR* fl/d3 polymorphism with BMI by region**

| City | Sex | Category | WT/HT/MT | Additive model | |  | Dominant model | |  | Recessive model | |
| --- | --- | --- | --- | --- | --- | --- | --- | --- | --- | --- | --- |
|  |  |  |  | OR (95% CI) | *P*-value |  | OR (95% CI) | *P*-value |  | OR (95% CI) | *P*-value |
| Yixing | Boys | Normal BMI | 633/274/36 | Reference |  |  | Reference |  |  | Reference |  |
|  |  | Low BMI | 221/93/16 | 1.036(0.830-1.294) | 0.753 |  | 1.003(0.768-1.310) | 0.985 |  | 1.300(0.710-2.381) | 0.395 |
|  |  | Overweight | 83/27/1 | 0.678(0.454-1.013) | 0.058 |  | 0.686(0.437-1.075) | 0.100 |  | 0.232(0.031-1.710) | 0.152 |
|  |  | Obesity | 93/30/7 | 0.909(0.649-1.274) | 0.579 |  | 0.809(0.539-1.212) | 0.304 |  | 1.452(0.631-3.340) | 0.380 |
|  | Girls | Normal BMI | 588/228/31 | Reference |  |  | Reference |  |  | Reference |  |
|  |  | Low BMI | 271/98/9 | 0.877(0.696-1.104) | 0.264 |  | 0.892(0.682-1.166) | 0.403 |  | 0.629(0.297-1.336) | 0.228 |
|  |  | Overweight | 66/23/4 | 0.964(0.647-1.435) | 0.856 |  | 0.924(0.577-1.481) | 0.743 |  | 1.160(0.400-3.361) | 0.785 |
|  |  | Obesity | 82/30/3 | 0.897(0.619-1.299) | 0.564 |  | 0.909(0.591-1.398) | 0.664 |  | 0.691(0.208-2.298) | 0.547 |
| Suqian | Boys | Normal BMI | 438/144/29 | Reference |  |  | Reference |  |  | Reference |  |
|  |  | Low BMI | 160/68/19 | 1.323(1.034-1.693) | 0.026 |  | 1.370(1.000-1.878) | 0.050 |  | 1.667(0.916-3.032) | 0.094 |
|  |  | Overweight | 35/10/1 | 0.777(0.429-1.407) | 0.405 |  | 0.792(0.393-1.595) | 0.514 |  | 0.444(0.059-3.338) | 0.431 |
|  |  | Obesity | 28/19/0 | 1.239(0.757-2.027) | 0.395 |  | 1.710(0.931-3.143) | 0.084 |  | - | - |
|  | Girls | Normal BMI | 342/140/7 | Reference |  |  | Reference |  |  | Reference |  |
|  |  | Low BMI | 174/64/12 | 1.148(0.859-1.533) | 0.351 |  | 1.020(0.732-1.422) | 0.906 |  | 3.457(0.944-8.895) | 0.110 |
|  |  | Overweight | 41/8/0 | 0.474(0.225-1.001) | 0.050 |  | 0.456(0.209-0.996) | 0.049 |  | - | - |
|  |  | Obesity | 32/11/1 | 0.929(0.502-1.722) | 0.816 |  | 0.876(0.439-1.748) | 0.707 |  | 1.595(0.192-13.264) | 0.666 |

WT, *GHR* fl/fl genotype; HT, *GHR* fl/d3 genotype; MT, *GHR* d3/d3 genotype; OR, odds ratio; CI, confidence interval.

| **Table S3. Stratification analysis of *GHR* fl/d3 polymorphism with hypertension by region** | | | | | | | | | | | |
| --- | --- | --- | --- | --- | --- | --- | --- | --- | --- | --- | --- |
| Region | Sex | Category | WT/HT/MT | Additive model | |  | Dominant model | |  | Recessive model | |
|  |  |  |  | OR (95% CI) | *P*-value |  | OR (95% CI) | *P*-value |  | OR (95% CI) | *P*-value |
| Yixing | Boys | Normotensive | 502/241/32 | Reference |  |  | Reference |  |  | Reference |  |
|  |  | Pre-hypertension | 219/75/9 | 0.750(0.585-0.962) | 0.023 |  | 0.706(0.527-0.945) | 0.019 |  | 0.703(0.332-1.491) | 0.358 |
|  |  | Hypertension | 309/108/19 | 0.828(0.669-1.025) | 0.082 |  | 0.757(0.587-0.976) | 0.031 |  | 1.047(0.586-1.870) | 0.878 |
|  | Girls | Normotensive | 642/218/20 | Reference |  |  | Reference |  |  | Reference |  |
|  |  | Pre-hypertension | 156/68/12 | 1.366(1.055-1.770) | 0.018 |  | 1.357(0.996-1.847) | 0.053 |  | 2.231(1.074-4.633) | 0.031 |
|  |  | Hypertension | 227/93/15 | 1.273(1.009-1.606) | 0.042 |  | 1.259(0.957-1.655) | 0.100 |  | 1.952(0.987-3.860) | 0.054 |
| Suqian | Boys | Normotensive | 543/203/43 | Reference |  |  | Reference |  |  | Reference |  |
|  |  | Pre-hypertension | 68/24/3 | 0.848(0.576-1.249) | 0.405 |  | 0.871(0.544-1.394) | 0.564 |  | 0.558(0.170-1.835) | 0.337 |
|  |  | Hypertension | 50/14/3 | 0.800(0.502-1.273) | 0.346 |  | 0.745(0.421-1.319) | 0.313 |  | 0.802(0.242-2.658) | 0.719 |
|  | Girls | Normotensive | 497/184/14 | Reference |  |  | Reference |  |  | Reference |  |
|  |  | Pre-hypertension | 44/20/4 | 1.449(0.930-2.258) | 0.101 |  | 1.378(0.815-2.328) | 0.231 |  | 2.991(0.956-9.356) | 0.060 |
|  |  | Hypertension | 48/19/2 | 1.116(0.696-1.791) | 0.649 |  | 1.105(0.645-1.894) | 0.717 |  | 1.429(0.318-6.420) | 0.642 |

WT, *GHR* fl/fl genotype; HT, *GHR* fl/d3 genotype; MT, *GHR* d3/d3 genotype; OR, odds ratio; CI, confidence interval.

| **Table S4. Stratification analysis of *GHR* fl/d3 polymorphism with metabolic traits by region** | | | | | | | | | | | |
| --- | --- | --- | --- | --- | --- | --- | --- | --- | --- | --- | --- |
| Region | Sex | Category | WT/HT/MT | Additive model* | |  | Dominant model* | |  | Recessive model* | |
|  |  |  |  | OR (95% CI) | *P*-value |  | OR (95% CI) | *P*-value |  | OR (95% CI) | *P*-value |
| Yixing | Boys | Normal group | 874/362/53 | Reference |  |  | Reference |  |  | Reference |  |
|  |  | High GLU | 156/62/7 | 0.919(0.708-1.194) | 0.528 |  | 0.929(0.683-1.264) | 0.639 |  | 0.757(0.339-1.693) | 0.498 |
|  |  | Normal group | 907/381/57 | Reference |  |  | Reference |  |  | Reference |  |
|  |  | High TC | 123/43/3 | 0.726(0.527-1.007) | 0.081 |  | 0.771(0.539-1.102) | 0.154 |  | 0.407(0.126-1.315) | 0.133 |
|  |  | Normal group | 872/370/53 | Reference |  |  | Reference |  |  | Reference |  |
|  |  | High TG | 158/54/7 | 0.821(0.626-1.076) | 0.153 |  | 0.792(0.577-1.088) | 0.150 |  | 0.771(0.346-1.719) | 0.525 |
|  | Girls | Normal group | 874/319/41 | Reference |  |  | Reference |  |  | Reference |  |
|  |  | High GLU | 133/60/6 | 1.119(0.849-1.475) | 0.424 |  | 1.189(0.858-1.647) | 0.299 |  | 0.891(0.370-2.144) | 0.797 |
|  |  | Normal group | 867/334/45 | Reference |  |  | Reference |  |  | Reference |  |
|  |  | High TC | 140/45/2 | 0.774(0.565-1.061) | 0.111 |  | 0.769(0.541-1.093) | 0.144 |  | 0.288(0.069-1.197) | 0.087 |
|  |  | Normal group | 843/329/39 | Reference |  |  | Reference |  |  | Reference |  |
|  |  | High TG | 164/50/8 | 0.870(0.659-1.148) | 0.325 |  | 0.811(0.587-1.121) | 0.205 |  | 1.121(0.517-2.433) | 0.772 |
| Suqian | Boys | Normal group | 638/234/47 | Reference |  |  | Reference |  |  | Reference |  |
|  |  | High GLU | 23/7/2 | 0.967(0.520-1.799) | 0.916 |  | 0.929(0.683-1.264) | 0.639 |  | 1.257(0.0.291-5.420) | 0.759 |
|  |  | Normal group | 621/231/47 | Reference |  |  | Reference |  |  | Reference |  |
|  |  | High TC | 40/10/2 | 0.738(0.427-1.274) | 0.275 |  | 0.671(0.346-1.298) | 0.236 |  | 0.737(0.174-3.122) | 0.678 |
|  |  | Normal group | 649/237/49 | Reference |  |  | Reference |  |  | Reference |  |
|  |  | High TG | 12/4/0 | 0.686(0.252-1.872) | 0.462 |  | 0.757(0.242-2.367) | 0.632 |  | - | - |
|  | Girls | Normal group | 571/215/20 | Reference |  |  | Reference |  |  | Reference |  |
|  |  | High GLU | 18/8/0 | 0.959(0.444-2.069) | 0.911 |  | 1.068(0.458-2.493) | 0.878 |  | - | - |
|  |  | Normal group | 541/208/20 | Reference |  |  | Reference |  |  | Reference |  |
|  |  | High TC | 48/15/0 | 0.688(0.393-1.202) | 0.189 |  | 0.732(0.402-1.334) | 0.308 |  | - | - |
|  |  | Normal group | 575/220/20 | Reference |  |  | Reference |  |  | Reference |  |
|  |  | High TG | 14/3/0 | 0.502(0.152-1.661) | 0.259 |  | 0.507(0.144-1.781) | 0.289 |  | - | - |

* adjusted for age; WT, *GHR* fl/fl genotype; HT, *GHR* fl/d3 genotype; MT, *GHR* d3/d3 genotype; OR, odds ratio; CI, confidence interval.

| **Table S5. Multiplicative interaction of *GHR* d3 polymorphism and gender on pre-hypertension** | | | | |
| --- | --- | --- | --- | --- |
| Variable | β | SE | *P*-value | OR (95% CI) |
| Gender | -0.433 | 0.102 | <0.001 | 0.648 (0.531-0.792) |
| *GHR* dominant | -0.804 | 0.282 | 0.004 | 0.448 (0.258-0.778) |
| *GHR* dominant×Gender | 0.551 | 0.182 | 0.003 | 1.735 (1.214-2.481) |

OR, odds ratio; CI, confidence interval; SE, standard error.

| **Table S6. Multiplicative interaction of *GHR* d3 polymorphism and gender on hypertension** | | | | |
| --- | --- | --- | --- | --- |
| Variable | β | SE | *P*-value | OR (95% CI) |
| Gender | -0.339 | 0.091 | <0.001 | 0.713(0.596-0.852) |
| *GHR* dominant | -0.628 | 0.255 | 0.014 | 0.534 (0.323-0.880) |
| *GHR* dominant×Gender | 0.412 | 0.162 | 0.013 | 1.509(1.092-2.086) |

OR, odds ratio; CI, confidence interval; SE, standard error.
